# The Beneficial Impact of Pulmonary Rehabilitation in Idiopathic Pulmonary Fibrosis: A Review of the Current Literature

**DOI:** 10.3390/jcm13072026

**Published:** 2024-03-30

**Authors:** Stefano Sanduzzi Zamparelli, Carmen Lombardi, Claudio Candia, Paola Rebecca Iovine, Gaetano Rea, Michele Vitacca, Pasquale Ambrosino, Marialuisa Bocchino, Mauro Maniscalco

**Affiliations:** 1 Division of Pneumology and Respiratory Physiopathology, A. Cardarelli Hospital, 80131 Naples, Italy; stefanosanduzzi@gmail.com; 2 Istituti Clinici Scientifici Maugeri IRCCS, Pulmonary Rehabilitation Unit of Telese Terme Institute, 82037 Telese Terme, Italy; carmen.lombardi@icsmaugeri.it; 3 Department of Clinical Medicine and Surgery, University of Naples “Federico II”, 80131 Naples, Italy; claudio.candia@unina.it (C.C.); marialuisa.bocchino@unina.it (M.B.); 4 Department of Radiology, Monaldi Hospital, Azienda Ospedaliera dei Colli, 80131 Naples, Italy; gaetano.rea@ospedalideicolli.it; 5 Istituti Clinici Scientifici Maugeri IRCCS, Pulmonary Rehabilitation Unit of Lumezzane Institute, 25065 Lumezzane, Italy; michele.vitacca@icsmaugeri.it; 6 Istituti Clinici Scientifici Maugeri IRCCS, Scientific Directorate of Telese Terme Institute, 82037 Telese Terme, Italy; pasquale.ambrosino@icsmaugeri.it

**Keywords:** idiopathic pulmonary fibrosis, pulmonary rehabilitation, outcome, disability, exercise, occupational medicine, cardiovascular outcomes, health-related quality of life

## Abstract

Idiopathic pulmonary fibrosis (IPF) is a chronic and irreversible fibrotic disease whose natural history is characterised by a progressive worsening of the pulmonary function, exertional dyspnoea, exercise intolerance, reduced physical activity, and health-related quality of life (HRQOL) impairment. Pulmonary rehabilitation (PR) is a comprehensive, multi-disciplinary programme that uses a combination of strength training, teaching, counselling, and behaviour modification techniques to reduce symptoms and optimise functional capacity in patients with chronic lung disease. Based on the well-documented effectiveness of PR in chronic obstructive pulmonary disease (COPD), over the years supportive evidence of its benefits for other respiratory diseases has been emerging. Although the latest rehabilitation guidelines recognised PR’s efficacy for interstitial lung disease (ILD) and IPF in particular, this comprehensive approach remains underused and under-resourced. In this review, we will discuss the advantages and beneficial effects of PR on IPF, analysing its impact on exercise capacity, disease-related symptoms, cardiovascular outcomes, body composition, and HRQOL.

## 1. Introduction

Idiopathic pulmonary fibrosis (IPF) is a rare, chronic, progressive, and irreversible fibrosing interstitial lung disease (ILD) of unknown aetiology with an unfavourable prognosis [1]. It is associated with a significant deterioration of pulmonary function up to chronic respiratory failure and death in the short term, with an estimated average survival from diagnosis of 2.5/3.5 years in untreated patients [1]. The clinical presentation is characterised by non-specific symptoms, such as dry cough and exertional dyspnoea, and variable clinical course. Therefore, either the diagnosis is delayed (often up to several years) or the disease is misdiagnosed as COPD, gastroesophageal reflux disease (GERD), or a heart condition [2]. In most cases (>60%), the disease presents an aggressive behaviour, with a constant decline in lung function and frequent episodes of accelerated progression (“exacerbations”), known to be highly lethal [3,4]. To date, pirfenidone and nintedanib are the only two approved and commercially available antifibrotic drugs (AFDs); their actions result in a significant slowdown of the disease’s progression [5,6], but the prognosis remains unsatisfactorily poor, and the burden of the disease is still unbearable for most patients. Finally, the coexistence of additional morbidities exerts a significant impact on the disease’s clinical course and health-related quality of life (HRQOL). Among these, pulmonary hypertension, coronary artery diseases, and lung cancer have the worst impact on disease survival [7]. The comprehensive care approach of IPF over time relies, therefore, on the pharmacological treatment in association with several non-pharmacological interventions, such as patient education, psychological and nutritional support, and pulmonary rehabilitation (PR).

PR is a combination of structured and progressive individually tailored exercise training, self-management education, and patient assessment delivered by a multidisciplinary team of healthcare professionals [8]. The latest ATS guidelines [9] have emphasised the importance of tailoring the PR programme according to the patient’s needs, with a vast personalisation of components and modalities. Therefore, the most updated PR approaches include not only exercise training with aerobic or resistance conditioning and respiratory therapy, but also nutritional and educational interventions, physiological support, and behaviour modification techniques to improve self-management [10,11]. Over the last 20 years, aerobic exercise schemes have shown different beneficial effects on patients with chronic respiratory diseases [12,13], improving not only the exercise capacity, but also health-related quality of life (HRQOL) and fatigue and dyspnoea scoring; accordingly, scientific evidence about the beneficial effects of PR in IPF has seen a significant increase [14,15]. The use of PR in the setting of IPF and other interstitial lung diseases (ILDs) has been long discussed in previous works [16,17,18,19,20], mostly systematic reviews and meta-analyses, which have underscored its beneficial and multidimensional effects. However, few of them were evaluated as methodologically flawless in a recent systematic review [21], which underlined the overall low quality of the available evidence concerning the impact of PR on IPF. Moreover, several studies have included patients with a more generic diagnosis of interstitial lung disease, which covers a wide range of pathologies characterised by different mechanisms and underlying causes. Finally, no previous review, however, focused specifically on aspects such as the relationship between PR and pharmacological strategies and the optimal PR scheme for IPF patients.

In light of the above, the aim of the current narrative review is to examine the available evidence on the impact of PR on IPF patients from a multidimensional perspective, with a particular emphasis on which are the most promising schemes and approaches in this clinical setting.

All the studies included in the current narrative review have been summarised in Table 1 (design, main results, and outcomes of each study) and Table S1 (features of the PR programmes involved in the study).

## 2. Can PR Improve Exercise Capacity, HRQOL, and Cardiovascular (CV) Outcomes in IPF Patients?

Several mechanisms have been linked to the genesis of exercise limitation in IPF patients. Jackson et al. [27] pointed out that exercise limitation in IPF patients might be due to inefficient ventilation (V_E_), represented by a marked elevation of the V_E_/VCO_2_ slope during workout. Such impairment could be caused by a progressive shift to an anaerobic metabolism, as reflected by the increase in the plasmatic levels of 15-F2-isoprostanes, lactates, and glutamate, with a subsequent increase in reactive oxidative species (ROS), which impair calcium metabolism and myofilament function in skeletal muscle cells. This ultimately results in a lack of physical endurance [37]. Moreover, 15-F2-isoprostanes are directly linked to the increase in pulmonary vascular resistance due to a direct vasoconstrictor effect [38].

There is also evidence that IPF patients experience a more severe exercise-induced peripheral oxygen desaturation than patients with ILDs other than IPF, with a lower nadir during the 6MWT [39]. Circulatory limitations are mainly due to the destruction of the capillary bed and the resultant pulmonary reactive vasoconstriction, leading to ventilation–perfusion mismatch and oxygen diffusion limitations [40]. In addition, some patients may experience an additive impaired gas exchange due to the development of pulmonary hypertension and cardiac dysfunction [41]. Although not considered the leading mechanism of limited exercise tolerance, ventilatory limitations affect IPF patients through the restriction of both static and dynamic volumes [42]. As postulated by Miki et al. [43], a dysregulation in exertional acidosis using ventilatory compensation may force IPF patients to stop exercising to reach a normal pH level, suggesting that hypoxemia was not the direct cause of exercise limitation. Peripheral muscle dysfunction, driven partly by physical deconditioning and partly by corticosteroids and immunosuppressive therapy-induced myopathy, plays a major role in exercise limitation. Patients who experience dyspnoea and fatigue commonly reduce their activity levels, leading to a vicious cycle of worsening exercise capacity and increasing symptoms [44]. In this context, PR can contribute to muscle reconditioning, thus breaking the chain of peripheral muscle dysfunction, dyspnoea, and deconditioning.

### 2.1. The Impact of PR on Exercise Capacity

Among the different measurements of outcome, the 6 min walking test (6MWT) has been broadly used in respiratory patients because it is easily tolerated by patients, it is easy to implement, and is an acceptable measurement of submaximal effort [45,46] as well as an independent predictor of mortality and disease progression [47].

Choi et al. [23] compared two groups of IPF patients, of which one underwent PR, with significant improvements in the 6MWD in the interventional group at the end of PR. Exercise programmes also improved the maximum oxygen consumption (VO_2_ max) measured by the cardiopulmonary test (CPET), an indicator of maximal exertion levels. This is known to be the best indicator of aerobic health, whose changes are associated with variations in mortality, and an independent predictor of all-cause deaths in healthy adults [48]. Consistently, Nishiyama et al. [49] reported that a 10-week exercise programme was able to significantly improve the performance of IPF patients in the 6MWT, with a mean post-PR variation (Δ) of 46.3 m, despite no relevant change in pulmonary function or dyspnoea.

However, Jacksons et al. [27] did not find any significant change in the 6MWT in IPF patients at the end of the 3-month PR programme but reported different physical and clinical benefits. In particular, at the end of PR the experimental group presented with an improvement in exercise tolerance assessed as endurance time at cycle ergometry, an improved respiratory muscle strength assessed by the maximal inspiration pressure (MIP), and a better stable exercise capacity assessed as oxygen uptake (VO_2_) during constant-load testing; in contrast, the control group experienced a significant decrement in such tests over the same time span.

The impact of PR in improving short-term exercise tolerance, pulmonary function, and disease-related symptom perception was further strengthened by Vainshelboim et al. [31]. Unlike previous studies, the increase in physical health following PR was confirmed by the enhancement in patients’ submaximal (anaerobic threshold and 6MWD) and maximal exertion (VO_2_ peak and peak work rate, WR) levels and leg strength assessed through the 30 s chair-stand test. Compared to the control group, the PR group showed a better functional capacity, with a Δ6MWD of 81 m and a ΔpeakWR of 22.1 W. IPF patients who underwent PR experienced a significant improvement in ventilatory parameters, such as forced vital capacity (FVC) and maximal voluntary ventilation (MVV). In line with Manali et al. [50], the increase in the exercise capacity resulted in a reduced perception of dyspnoea during exercise and an amelioration in HRQOL. This improved aerobic capacity was apparently secondary to a better ventilatory function, represented by a higher VO2 peak, thanks to an increase in alveolar oxygen tension and alveoli ventilation-to-perfusion mismatch. The significant correlation (r = 0.775, *p* = 0.001) found by the authors between the variation in VO_2_ peak and peak tidal volume further supports this hypothesis. Finally, such data suggest that skeletal muscle and cardiovascular adaptations could contribute to some extent to the increase in exercise tolerance, as evidenced by the improvement in the 30 s chair-stand test and peripheral oxygen (O_2_) saturation (SpO_2_).

Finally, Arizono et al. [22] compared the responsiveness of different exercise measurements in IPF patients upon PR. Besides 6MWD, peak VO_2_, peak WR, endurance time (ET), and incremental shuttle walking distance (ISWD) were evaluated. The authors concluded that ET is likely the best-performing assessment, similarly to findings in COPD subjects [51,52]. In fact, ET measures the ability to sustain submaximal exercise capacity, with an improvement even in the absence of a significant increase in maximum exercise capacity [53]. According to the authors [22], a constant-load exercise test reflects the severity of dyspnoea better than a test of peak exercise performance such as 6MWT due to the similarity of the effort of the constant-load exercise test to daily activity efforts. Based on the correlation between ET and anaerobic threshold changes, the authors hypothesised that the improvement in ET could be linked to the reduction in exercise-induced lactic acidosis and the incrementation of oxidative enzymes in the peripheral muscles. Compared to COPD subjects, in IPF patients the improvement in ET seemed not to be influenced by the improvement in muscle strength and could rather be related to variations in the oxygen delivery.

Overall, however, there is evidence of the effectiveness of PR in the setting of IPF, with widely confirmed improvements in several outcome variables such as 6MWD and ET.

### 2.2. PR and Quality of Life in IPF

HRQOL represents the subjective perception of the impact of physical and mental health status on quality of life [54]. Improving HRQOL has been widely recognised as an important feature in the rehabilitative management of chronic diseases, especially COPD, since compromised physical health and depression impact negatively on disease perception and dyspnoea [55]. Although HRQOL has been studied for many chronic respiratory diseases, few studies have evaluated its impact on IPF. HRQOL can be assessed by standardised and validated questionnaires such as the SF-36 and the SGRQ, which evaluate the patients’ perceived health and symptoms [56]. The link between impaired HRQOL and decreased physical function was underscored by Tomioka et al. [57], who showed that the worse the levels of SF-36, the lower the vital capacity and the 6MWD. Multiple evidence [10,58] suggests that physical training in the form of PR minimises IPF-related symptoms and produces a marginal improvement in HRQOL immediately after training, with no beneficial effect in the long term.

In this context, Gaunard et al. [25] investigated the impact of a 3-month PR programme on increasing the endurance and strength of IPF patients and their HRQOL, assessed using a specific version of the SGRQ (SGRQ-I) more focused on the subjective wellbeing of IPF patients and response to exercise training, while daily physical activity was assessed through the International Physical Activity Questionnaire (IPAQ). This study reported a significant increase in physical activity measured by IPAQ and in HRQOL evaluated by SQRQ-I. Consistent with the previous literature [50], the PR group reported a significant decrease in self-reported physical activity during the follow-up, showing a substantial reversal of the results of PR on physical activity and the need for a long-lasting PR programme, or multiple cycles of PR, to maintain a high level of physical activity.

Finally, Jarosh et al. [28] observed that IPF patients who underwent a PR programme had a significant change in the Chronic Respiratory Questionnaire (CRQ), with a ΔCRQ of 3.0 at the end of the programme. The authors also observed that patients with a preserved FVC or with high anxiety levels assessed by the hospital anxiety and depression scale (HADS) had the best likelihood of benefitting from PR, and these two parameters were found to be the best predictors of PR success in the short term. Although still limited evidence is available, it appears that PR is able to induce improvements in HRQOL in IPF patients, thus reducing, at least partially, the burden of the disease.

### 2.3. Pulmonary Rehabilitation, Cardiovascular Outcomes, and Body Composition in Idiopathic Pulmonary Fibrosis

Coronary artery disease (CAD), pulmonary hypertension, and right ventricle dysfunction (RVD) are highly prevalent comorbidities in subjects with IPF, resulting in a worse prognosis and in an increased rate of complications and mortality [59]. The amelioration of exercise tolerance and aerobic capacity due to PR, as well as the changes in lifestyle and nutrition, might lead to the improvement of the cardiovascular function, with beneficial effects on the prognosis.

Vainshelboim et al. [34] analysed a small cohort of 32 IPF patients and found significant changes in exercise cardiovascular indices (such as peak circulatory power, peak cardiac power, peak stroke work, VO_2_ peak_,_ intra-ventricular septum thickness, and left ventricle end-diastolic diameter index) between those who underwent PR and the controls. In IPF subjects, this increase in peak VO_2_ could be mainly attributed to the increase in the cardiac output. In addition, improvements in respiratory mechanisms may also play a significant contribution to the improvement in the peak VO_2_, as previously demonstrated by the significant correlation between ∆peak tidal volume and ∆peakVO_2_ in the PR group [31]. Moreover, the beneficial impact of the enhancement in exercise cardiovascular function on the prognosis was emphasised by the significant correlations between the variations in the exercise cardiovascular indexes and exercise capacity, dyspnoea, and HRQOL (Table 1). This improvement in peak oxygen consumption due to aerobic exercise training relies on the enhancement in oxygen diffusion at the alveolar level, as well as the increase in oxygen extraction in peripheral tissues.

In line with the previous findings, Vainshelboim et al. [33] demonstrated the short-term beneficial impact of participating in a PR programme on enhancing physical activity levels and body composition in IPF patients, although those benefits were not sustained over time. Compared to the control group, who experienced a progressive deterioration during the observational time span, patients undergoing PR increased their physical activity levels assessed by IPAQ and significantly reduced their waist circumference, weight, and body fat, the latter considered to be a major cardio-metabolic risk factor [59]. Data derived from the abovementioned studies support the hypothesis that PR can affect cardiovascular outcomes positively, mostly thanks to the action on body composition. Therefore, targeting obesity is crucial in order to improve exercise capacity, lung function, and cardiovascular risk.

## 3. How Long Do the Effects of PR Last in IPF Patients?

Only a few studies have evaluated the persistence of the beneficial effects of PR in IPF patients over time. Ryerson et al. [60] studied a group of 39 ILD patients (17 patients with IPF) who completed a PR programme and reported a stable Δ6MWD of 49.8 m at a 6-month follow-up. Following these observations, Jarosch et al. [28] analysed the maintenance over time of PR-related benefits in an IPF cohort. The authors found that the PR impact on exercise capacity was not as durable over time compared to the medium-term maintenance of HRQOL improvement. Also, the initial significant improvement in the 6MWD (∆6MWD = 61 m) at the end of PR did not reach statistical significance at the 3-month follow-up (∆6MWD = 26 m), although the result was within the range of clinical relevance (minimal clinically important difference, MCID: 25–33 m). The authors assumed that the duration of PR may be an issue since longer programmes may influence patients’ behaviour by promoting a more active lifestyle and reducing anxiety-related symptoms, both helpful elements to maintain their exercise performance. Regarding HRQOL, the improvement in the Chronic Respiratory Questionnaire total score between both groups immediately after completing PR (∆CRQ = 3.0) was maintained up to the 3-month follow-up (∆CRQ = 3.5). This might be explained by the patients’ growing disease-specific knowledge and their skill to manage dyspnoea with better breathing strategies thanks to educational programmes. The advantage of having a better knowledge of IPF disease is confirmed by the improvement in SF-36 mental component summary score in a short time compared to the control group (∆SF-36 = 7.1).

In an attempt to address the long-term effects of PR in IPF patients, Vainshelboim et al. [32] reported that at 11 months of follow-up after PR, only leg strength (assessed through the 30 s chair stand test) and HRQOL (assessed through the SGRQ) were preserved. However, compared to the deterioration trend that characterised the non-interventional group, patients who underwent PR maintained stability in most of their baseline outcomes. These findings are probably linked to the “detraining effect”, according to which the gradual reversion of the acquired adaptation to training comes as a consequence of the removal of the physiological stimulus that drove the adaptation, according to the “principle of reversibility” [42]. In addition, part of this worsening in clinical outcomes could be explained by the progressive natural history of IPF [61]. It is likely that some physiological and subjective–perceptual adaptations to PR, such as muscle strength and HRQOL, deteriorate slower than other clinical outcomes among IPF patients, resulting in better preservation of these parameters. Finally, the association between 6MWD changes and HRQOL (r = −0.82, *p* < 0.001) pointed out by this study [32] raised doubts about the weight of walking capacity in determining health in IPF patients. These results further emphasise the significance of maintaining PR short-term improvements by implementing a continuous long-term programme for IPF patients. Those data are consistent with Holland et al. [62], who demonstrated unsustained benefits in most of the clinical outcomes at a 6-month follow-up after PR.

In the end, most of the beneficial effects of PR in IPF patients tend to vanish within a range of 3–6 months from the end of the programme, even if HRQOL seems to maintain a certain degree of stability. This phenomenon might be due to several factors, including the disease’s clinical evolution, which is known to be progressive. A possible approach to this problem, however, might be considering patients for at least two cycles of intensive inpatient or outpatient supervised PR, followed by home-based exercise programmes. This could, in fact, maximise the beneficial effects and help maintain them throughout the whole year. Of course, this proposal should be validated by appropriately designed clinical trials.

## 4. Do PR and AFDs Have a Synergistic Effect When Combined?

Since their introduction into clinical practice, the two AFDs, pirfenidone and nintedanib, have represented the cornerstone of the pharmacological therapy of IPF. Both contribute to slowing down the decline in lung function and reduce the frequency of hospitalisation and acute exacerbation, although with different molecular mechanisms [5,6]. However, these therapies are not free from side effects, such as gastrointestinal (nausea, diarrhoea, anorexia, and liver dysfunction) or cutaneous (photosensitivity and rashes), mainly responsible for the rate of discontinuation of therapy. Moreover, such side effects can impair HRQOL, exercise tolerance, and daily life. Considering the available evidence [29,50], which shows an increase in 6MWD and HRQOL and a reduction in dyspnoea following PR, therapy with AFDs should be integrated by non-pharmacological programmes, especially PR. To date, only a few studies have focused on the effects of the combination of AFDs and PR.

Iwanami et al. [26] described a significantly higher value of Δ6MWD in patients who received both PR and AFD when compared to only AFD-treated cases (PR + AFD: + 34.1 m; AFD: −34.5 m) and inferred a synergistic effect of PR and AFDs. This could be explained by the capacity of PR to prevent the decline in 6MWD and HRQOL caused by the side effects of AFDs, although the overall incidence of side effects has been reported to be similar in treated and untreated patients [63]. The negative impact of AFD-related side effects on the patient’s health status is confirmed by the progressive worsening of CAT as a measure of HRQOL in treated patients. Furthermore, this study, according to previous findings [14], confirmed the effect of PR in increasing the exercise capacity expressed as 6MWD and the dyspnoea severity expressed as modified British Medical Research Council Questionnaire (mMRC). The close relationship between the subjectivity of perceived symptoms and exercise tolerance was confirmed by the finding of a significant negative correlation between ∆mMRC and ∆6MWD/∆6MWD% (r = −0.337, *p* < 0.05 and r = −0.331, *p* < 0.05, respectively), and between ∆6MWD/∆%6MWD and ∆SGRQ (r = −0.277, *p* < 0.05 and r = −0.301, *p* < 0.05, respectively). In this way, amelioration of the 6MWD was partly dependent on the improvement of dyspnoea.

The FITNESS study [29] was the first RCT to evaluate the long-term effects of a combination of outpatient and home-based PR schemes in IPF patients taking AFDs. Although no significant difference in 6MWD was found between the two groups after completing the 52-week training period, the change in ET using a cycle ergometer was significantly better in the PR group than in the control one, with a ∆ET of 187 s. This result confirms the importance of a multidisciplinary approach to IPF patients and underscores the importance of PR as a therapeutical asset to improve the wellbeing of patients. Further research should corroborate the previous findings in order to cast light on the relationship between AFDs’ side effects and exercise capacity and on the strategies to prevent or limit them.

## 5. Which Are the Most Appropriate Settings and Programmes for PR in IPF Patients?

Although most of the PR programmes for IPF patients are offered in hospitals, not all patients are suitable candidates due to severely compromised mobility, the long traveling distance to rehabilitation services, and the high risk of infection [47]. Consequently, interest in tele-rehabilitation is growing as an alternative to the usual outpatient PR programmes [64]. Based on the more flexible home-based setting, tele-rehabilitation ensures participation where and whenever the patients find it suitable, thus overcoming the problem of distance and transfer. There is evidence that tele-rehabilitation is as effective as a standard rehabilitation programme in chronic cardiopulmonary diseases like bronchial asthma and chronic heart failure [65].

In the setting of IPF, Cerdàn-de-las-Heras et al. [24] analysed the impact of a virtual autonomous physiotherapist agent (VAPA), a new platform for tele-rehabilitation proposed as an alternative to the standard of care, on a group of 15 IPF patients, who were compared to a control group. According to the authors, VAPA can ensure online participation in PR sessions, thanks to the digitalisation of the training scheme, including aerobic exercise and respiratory muscle training with nutritional, physiological, and educational intervention due to e-learning packages. Despite the social benefits experienced in conventional outpatient group training, which could not be transferred to a video training set-up, tele-rehabilitation allows patients to experience training at home, without the fixed schedule of traditional home-based PR. This PR attitude seems to be preferred by the patients, with a reported high adherence that continued to increase even in the follow-up period with more frequent and longer work-out sessions, despite a decrease in the number of participants over time (0–3 months: 15 patients had a 64% adherence with a 16.5 min training; 3–6 months: 5 patients had a 108% adherence with a 19.5 min training; 6–9 months: 3 patients had a 110% adherence with a 21 min training). Cerdàn-de-las-Heras also showed that, compared to the control group, which experienced a continuous deterioration in 6MWD as time advanced, VAPA maintained the patients’ exercise capacity measured as 6MWD at 3 (+39.5 m) and 6 months (+34.3 m) from baseline.

Following the positive results obtained in COPD patients [66], Yuen et al. [35] analysed the effectiveness of placing exergames into a home-based exercise programme in improving functional performance and quality of life in subjects with IPF. The term exergaming refers to technology-driven physical activities, such as video game play, that require participants to be physically active to play the proposed game, which becomes a real tool for physical activity [67]. Despite the promising premises, however, the authors did not find any improvement in physical function, HRQOL, and exercise-related dyspnoea following this PR intervention, maybe due to the low adherence rate (reported to be around 20%). Nonetheless, those patients who underwent PR with exergames experienced a progressive deterioration in both 6MWD and SGRQ from baseline to post-intervention, as well as the control group. Those data highlighted the importance of a close behavioural monitoring system of exercises over time.

The interventional studies analysed show a huge variability in the exercise training protocols used for the PR programmes in IPF patients (aerobic, resistance, stretching, and balance training). Moreover, PR can take place in different settings, thus affecting the adherence degree of the patient as well as the overall effectiveness of the therapy. Most of the studies so far included in this review took place in a supervised outpatient [22,23,25,27,31,32,33,34,49] or inpatient [28] setting, while only a few were conducted as home-based [24,33,35] or combined [26,29] PR programmes. Home-based PR is a self-care-based, mostly unsupervised intervention in which the physiotherapist provides a training scheme and the participants perform the PR exercise by themselves. The flexible schedules in terms of time and place allow patients to practice PR whenever they want and everywhere, thus leading to participation in patients who live long distances away.

Supervised outpatient PR programmes seem to guarantee a more significant gain in physical performance. The lower level of improvement of home-based and combined exercise training programmes could be justified by the lower adherence and the lack of control, with significantly fewer patients completing PR. Unlike other forms of ILDs, the increased degree of functional impairment of IPF patients requires a greater degree of care by healthcare personnel, indicating outpatient PR as a mode of preferred rehabilitation. Although reference guidelines emphasise the need for a PR programme in the holistic management of IPF patients, current lung rehabilitation focuses on a COPD-based whole-body physical exercise, which is associated with a high oxygen consumption rate [68].

Despite the well-known differences of the underlying mechanisms of dyspnoea in COPD and IPF, this rehabilitation scheme remains the most widely used in studies aimed at analysing the impact of training on exercise tolerance and HRQOL in IPF. Nowadays, is still not known which kind of specific exercise training might be the best to enhance IPF health status. The “LHP Respiratory Rehabilitation for Pulmonary Fibrosis” (LHP’s RRPF) designed by Li Shen et al. [30] was the first attempt to develop a PR programme based on the IPF lung characteristics, such as reduced lung tissue elasticity, limited lung expansion, decreased lung volume, reduced vital capacity, and growing hypoxia due to impaired gas exchange. LHP’s RRPF breathing exercises include three consecutive sets of movements repeated over time, affecting first the whole lung, then the unilateral lower and upper lung segments. Focusing only on exercising the respiratory muscles by slow deep breathing movements and minimising the involvement of other muscles, LHP’s RRPF practiced for 1 year was proved to reduce oxygen consumption. During the 6th month of the trial, the PR group showed a delayed lung function decline, highlighted by the significantly reduced ΔFVC rate decline (−0.007 vs. −0.155) and ΔFEV_1_ rate decline (−0.008 vs) compared to the control group. Furthermore, patients who underwent LHP’s RRPF maintained lung elasticity and experienced an increase in oxygen diffusion, expressed by a slightly positive ΔDLCO, compared to a reduction in the control group. Although there was no significant difference in the 6MWD at the 6-month timepoint, patients who practiced LHP’s RRPF experienced a significative improvement in HRQOL, pointed out by an average negative change in ΔSGRQ when compared to the control group. At the end of the trial, such differences were maintained. Moreover, patients who practiced LHP’s RRPF, besides continuing to preserve improvement in HRQOL assessed as ΔSGRQ, experienced over time a significant improvement in exercise endurance.

Based on the beneficial effects reported for pulmonary Dayoin (PD) in COPD patients [69], Zhou et al. [36] investigated its impact on subjects with IPF. PD represents an ancient Chinese mind–body technique similar to Taichi that combines a series of physical movements, breathing exercises, and respiratory muscle training. This study demonstrated that the PD programme was able to increase exercise capacity assessed by 6MWD; its effects lasted for at least 4 months after the end of the PD programme. Compared to the control group, PD is non-inferior to classical rehabilitation schemes in reducing disease-related breathlessness evaluated by mMRC following up for 4 months. Surprisingly, people who underwent PD presented a significant increase in FVC at 2 months. According to the reduction in lung compliance and the ineffectiveness of breathing patterns that occur in patients with IPF, the PD enhancements might be related to an improvement in breathing efficiency and muscle strength due to specific limb and trunk movements that expand the chest. Those findings encourage finding low-cost and easier substitutes for traditional PR in a comprehensive intervention for IPF patients.

Exercise training is a core component of PR and consists mainly of aerobic regimens, including endurance, resistance, and flexibility training. Based on the absence of a specific PR protocol for ILD patients, the American College of Sports Medicine (ACSM) Guidelines for Exercise Testing and Prescription [70] can be adopted in this class of patients. The exercise training principles derived from those successfully used in COPD patients should be carefully applied to ILD subjects because of their higher severity of exertional dyspnoea, lower level of exercise-induced desaturation, and rapid disease progression [58].

Endurance training, practiced by walking on a treadmill and/or biking on a stationary cycle ergometer, aims to improve aerobic capacity, exercise endurance, and physical activity. Regardless of the training method, it is prescribed at a frequency of 3–5 times per week of a 20–60-min session, with an intensity usually set at >60% of maximum exercise capacity, such as the walking speed on baseline 6MWT for walking exercise or peak work rate on CPET for cycling. Resistance training improves local muscle strength and endurance, and it can be practiced against gravity, body weight, or free weights. ACSM recommends training 2–3 days per week, with one to three sets of eight to twelve repetitions. For intensity, an initial load equivalent to the 60–70% of the maximal load that can be moved once over the full range of motion or one that induces fatigue after 8 to 12 repetitions is suggested, with an increasing exercise intensity over time. Flexibility training aims to increase the motion range of joints and muscles. Despite no studies confirming its contribution to ILD, the ACSM recommends flexibility exercise at least 2–3 days per week. Based on the frequent involvement of the upper extremities in everyday activity, upper limb training targeting the biceps, triceps, and deltoids is typically integrated into an exercise regimen. This kind of exercise can be achieved by either aerobic regimens such as arm cycle ergometer training or resistance training using free weights or elastic bands. A progressive increase in exercise should be tailored depending on the individual performance status of participants [70].

Recommended training variables such as the ideal frequency, intensity, time, and type (known as the FITT components) have not been defined yet for IPF. In most of the studies reported in this review (Supplemental Table S1), exercise training was practiced with an overall frequency of 2–3 days per week at a moderate-to-vigorous intensity [22,23,24,25,26,27,29,31,32,33,34,35,49], although a higher frequency at the same level of intensity was also reported to be beneficial [28,29,30,36]. Aerobic exercises had an average length of 20–30 min per session, with an intensity of over 60–80% maximal work rate delivered. For endurance training, the exercise modalities most commonly used in PR were walking on a treadmill and cycling using a continuous endurance training protocol, although interval training has also been used. Nonetheless, it is crucial to highlight that no significant differences have been observed between the physical activities in each protocol; therefore, most differences in the outcomes presented here might depend on the study design or study population, rather than on the type of physical activity in which patients were involved. Notably, studies conducted by Vainshelboim, Shen, and Zhou [29,31,36] have demonstrated that aerobics and breathing exercises are the most effective ones in improving lung function parameters and/or delaying pulmonary decay; therefore, clinicians might opt for such kind of activities whenever feasible.

To sum up the available evidence, it seems to be recommendable to incorporate a mixture of exercise paradigms, combining both inpatient/outpatient and home-based stages in order to achieve optimal results. The first phase should involve supervised activity that is tailored to the individual’s capacity and guided by healthcare professionals. During this phase, patients should learn how to perform the exercises correctly and safely, including aerobic, resistance, flexibility, balance training, and respiration techniques, and should be carefully instructed about self-monitoring techniques. The presence of a physiotherapist and constant monitoring of oxygen saturation levels and heart rate are vital factors in determining the patient’s physical capabilities and tailoring the exercise accordingly. This personalised approach might help patients feel more confident and less fearful when exercising without supervision, determining their adhesion to exercises and, therefore, overall PR success. Subsequent cycles of home-based activity should then be employed to maintain the benefit for a longer time span.

It is imperative, however, to integrate lifestyle modifications that foster physical activity and attend educational sessions to comprehend the significance of exercise, depression management, and symptom control. The combination of these approaches is vital to prolonging and maintaining the progress achieved through PR and creating a sustainable exercise routine at home or in community centres, thereby augmenting the long-term benefits of PR. In addition, social interaction with other patients represents a motivational factor that helps complete the programme successfully.

## 6. Future Directions of PR in IPF

So far, PR has been mostly employed in the COPD setting and its schemes have been developed accordingly. While it is true that COPD and IPF significantly differ from a physio-pathological point of view, they still share some elements, such as the impaired lung mechanics, the increased work of breathing, and the abnormal gas exchange, all contributing to worsen the HRQOL. The similarities have therefore represented the precondition for the use of PR outside COPD and pushed the scientific community to address the topic, also emphasised by the recommendation of PR for IPF patients since the 2011 ATS/ERS/JRS/ALAT guideline for IPF [61].

However, it is clear that novel and specifically designed PR schemes should be developed and validated in the near future in order to maximise the beneficial effects of exercise training and, if possible, achieve more durable results. In particular, home-based rehabilitation programmes have sparked interest in recent years, given the positive impact on transportation costs and logistical problems. Nonetheless, they seem to be affected by a lack of adherence in comparison to supervised outpatient training, which continues to represent the most effective setting for IPF patients. In fact, adherence to training is crucial in order to achieve the maximal improvements in physical performance and HRQOL, and the maximal adherence can be guaranteed by a stricter patient control by healthcare personnel in a dedicated facility. Novel PR schemes should consider adherence as a major limitation, and therefore, might endeavour to apply technologies, such as augmented reality glasses, in order to create a more immersive home-based setting for the patient. Offering on-demand resources, such as educational videos or web-based calls to specialists, might also improve adherence and build a more self-conscious approach to rehabilitation. Finally, healthcare professionals should be more often reminded of the importance of rehabilitation for IPF patients, especially through educational events.

Finally, clinical trials that investigate the role of gender and mental health status on the outcomes of PR in IPF patients are deemed necessary in order to achieve a more comprehensive depiction of the disease.

## 7. Conclusions

Considering the extensive amount of data that highlight the benefits of exercise training in IPF, PR should be recommended as the standard of comprehensive care for those subjects, as stated in the latest ATS rehabilitation guidelines [8].

**Figure 1:**
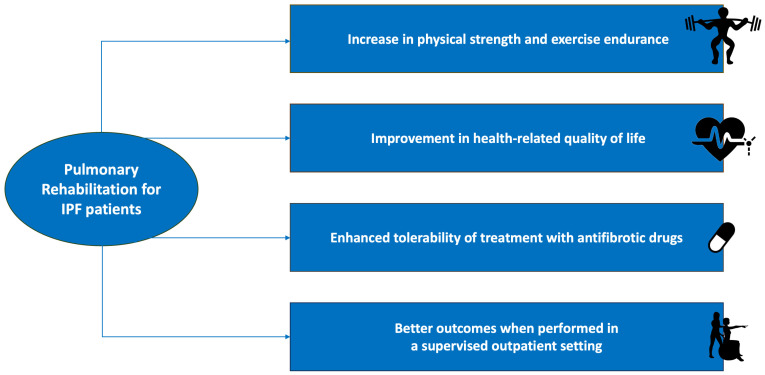
Summary of the beneficial effects of pulmonary rehabilitation in the clinical setting of idiopathic pulmonary fibrosis.

IPF patients who undergo PR programmes, in fact, do experience a significant improvement in exercise capacity, dyspnoea, HRQOL, and cardiopulmonary endurance in the short term (Figure 1); however, it is followed by a reprise of the physical decay just months after the end of the programme. Nonetheless, despite a certain degree of heterogeneity, it would seem that the improvements in dyspnoea and HRQOL may be sustained for a longer time span. In light of the above, further studies are needed to investigate techniques to promote an extension of the duration of those benefits. Nonetheless, the reportedly short permanence of the effects of PR might be at least partly due to the disease’s natural history, which is characterised by a progressive clinical decline.

Finally, since PR for IPF patients is still mostly based on programs designed for COPD patients, further efforts should be made to identify the most suitable programme in order to provide the greatest benefits of PR in patients affected by IPF.

## Figures and Tables

**Table 1:** Summary of the studies included in the current review.

Study	Study Type	IPF Subjects	Main Findings
Arizono, 2014 [22]	OS	48 total: 24 PR 24 CT	PR vs. CT: ↑ET, ↑peak WR, ↑AT, ↑work efficiency, ↑6MWD, and ↑ISWD
Choi 2023 [23]	OS	25 total: 13 PR 12 CT	PR vs. CT: ↑VO_2_ max, ↑VE/VCO_2_ slopes, ↑HR max, and ↑RER at VO_2_ max
Cerdán-de-las-Heras, 2021 [24]	RCT	29 total: 15 PR 14 CT	PR vs. CT: ↑∆6MWD
Gaunard, 2014 [25]	RCT	21 total: 11 PR 10 CT	PR vs. CT: ↓∆SGRQ-I and ↑∆IPAQ
Iwanami, 2022 [26]	OS	87 total: 29 PR 11 PR +AFD 26 CT 21 AFD	PR vs. AFD: ↑∆6MWD, ↑∆6MWD%, ↑∆mMRC PR+AFD vs. AFD: ↑∆6MWD PR vs. AFD: ↑∆mMRC PR group (pre- vs. post-PR): ↓mMRC, ↑ 6MWD and ↑6MWD%
Jackson, 2014 [27]	RCT	21 total: 11 PR 10 CT	PR vs. CT: ↑ET, ↑MIP, ↓SpO_2_, ↔6MWD ↑VO_2_ during exercise
Jarosch, 2020 [28]	RCT	44 total: 34 PR 17 CT	PR vs. CT: ↑∆6MWD, ↑∆ CRQ total score, ↑∆ SF-36 mental component summary score Between-group change in ∆CRQ total score persists after 3-month follow-up.
Kataoka, 2022 [29]	RCT	74 total: 38 PR 36 CT	PR vs. CT: ↑∆ET Between-group ∆6MWD last until the 26th week.
Shen, 2021 [30]	RCT	82 total: 39 PR 43 CT	6th month PR vs. CT: ↓FVC decay, ↓FEV_1_ decay, ↓ΔDLCO decay, ↓SGRQ 12th month PR vs. CT: all results confirmed
Vainshelboim, 2014 [31]	RCT	32 total: PR 15 CT 17	PR vs. CT: ↑FVC, ↑ΔMVV, ↑ΔPeak WR, ↑Δ6MWD ↑Δ30 s chair stand, ↓mMRC, ↓SGRQ
Vainshelboim, 2015 [32]	RCT	32 total: PR 15 CT 17	11th month PR vs. CT: ↑Δ30 s chair stand, ↓SGRQ In the PR group, a significant association between ΔSGRQ total score and Δ6MWD changes (r = −0.82, *p* < 0.001).
Vainshelboim, 2016 [33]	RCT	32 total: PR 15 CT 17	PR vs. CT: ↑ΔIPAQ, ↓waist circumference, ↓body fat Correlation between ∆IPAQ and ∆body fat (r = −0.496, *p* = 0.06)
Vainshelboim, 2017 [34]	RCT	32 total: PR 15 CT 17	PR vs. CT: ↑Δpeak circulatory power, ↑Δpeak cardiac power output, ↑ΔVO_2_ peak, ↑ΔHRR In the PR group, a correlation between ∆circulatory power and ∆6MWD (r = 0.66, *p* = 0.008), ∆circulatory power and ∆mMRC (r = −0.53, p = 0.042).
Yuen, 2019 [35]	RCT	20 total: PR 10 CT 10	PR vs. CT: ↔ 6MWD, ↔ SGRQ
Zhou, 2021 [36]	RCT	94 total: PD 32 PR 31 CT 31	2nd month PD vs. CT: ↑∆6MWD, ↓SGRQ, ↓mMRC, ↑FVC 4th month PD vs. CT: ↑∆6MWD, ↓mMRC 2nd month PD vs. PR: ↑∆6MWD 2nd month PR vs. CT: ↑∆6MWD, ↓mMRC 4th month PR vs. CT: ↑∆6MWD

↑ = significantly increased vs. control group (*p* < 0.05); ↔ = no significant difference vs. control group (*p* > 0.05); ↓ = significantly reduced vs. control group (*p* < 0.05). Abbreviations: RCT, randomised controlled trial; OS, observational study; PR, pulmonary rehabilitation group; CT, control group; VO_2_, peak oxygen consumption; WR, work rate; ET, endurance time; 6MWD, 6 min walking distance; ISWD, incremental shuttle walking distance; SpO_2_, peripheral oxygen saturation; HRR, heart rate at recovery; RER, respiratory exchange ratio; V_E_/VCO_2_ slopes, ventilation/carbon dioxide production slope; HR, heart rate; IPAQ, International Physical Activity Questionnaire; AFD, antifibrotic drugs; mMRC, modified British Medical Research Council Dyspnoea scale; SGRQ, St. George’s Respiratory Questionnaire; CRQ, Chronic Respiratory Questionnaire; FVC, forced vital capacity; FEV_1_, forced expiratory volume in 1 s; DLCO, diffusing capacity of the lungs for carbon monoxide; MVV, maximal voluntary ventilation.

## Data Availability

Not applicable.

## Supplementary Materials

The following supporting information can be downloaded at: https://www.mdpi.com/article/10.3390/jcm13072026/s1, Table S1: Setting, length, frequency of intervention, and protocols of the studies analysed.
