# Supplementary material for: The Beneficial Impact of Pulmonary Rehabilitation in Idiopathic Pulmonary Fibrosis: A Review of the Current Literature
Source: J Clin Med. 2024 Mar 30;13(7):2026. doi: 10.3390/jcm13072026 (PMC11012394; doi:10.3390/jcm13072026)
Supplement: Supplementary file 1 [file jcm-13-02026-s001.zip › jcm-2925859-supplementary.pdf]

Table S1. Setting, length, and frequency of intervention, and protocols of the studies analyzed.

STUDY	SETTING	LENGTH OF INTERVENTION	FREQUENCY	PR PROGRAM
**Arizono, 2014 [22]**	Outpatient	10 weeks	2/weekly	Supervised 90-min sessions of 15-min respiratory care (using an inspiratory threshold device with a 30% resistance of MIP) + subject education + 20-min endurance (continuous cycling at 80% of the peak WR) + strength training (upper and lower limb).
**Choi, 2023 [23]**	Outpatient	8 weeks	3/weekly	Sessions of 10-min breathing retraining and chest expansion exercise (using a threshold device) + 47-min aerobic exercise (interval training composed of 10-min warm-up at 50-70% of HRR, five 3-min intervals of treadmill walking at 70-85% of the HRR, four 3-min walks at 50-70% of the HRR, and a 10-min cool down at 50-70% of the HRR) + 10-min resistance exercise (upper and lower extremity).<br>The resistance exercises were performed at home repeatedly.
**Cerdán-de-las-Heras, 2021 [24]**	Tele-rehabilitation	12 weeks	3-5/weekly	VAPA is a (a) multidimensional software of tele-rehabilitation programs that unify video consultation sessions (planning with the physiotherapist the PR program) + e-learning packages (addressing psychological, nutritional, and medical aspects of IPF) + 10-20 min physical exercise programs (using elastics and weights) + online questionnaires (reporting satisfaction and adverse events) + patient digital files + real-time chat function (interacting whit the physiotherapist) and (b) a mobile app connected to a biometric sensor attached to the body.
**Gaunard, 2014 [25]**	Outpatient	12 weeks	2/weekly	Supervised 90-min sessions of 30-min aerobic exercises (20-min of treadmill walking and 10-min of cycling at 70–80% of the maximum HR) + 20-min flexibility exercises + 25-min strength training (upper and lower body using elastics) and 10 educational lectures provided throughout the program (including medication use, breathing techniques, exercise training, nutrition, pulmonary physiology, and psychological mechanism).<br>A home exercise program was practiced twice a week when they did not do PR.

**Iwanami, 2022 [26]**	Combined: supervised and home-based	12 weeks	1/weekly outpatient session<br>2-3/weekly home-based training	Outpatient supervised 60-min session of 20-30 min aerobic exercise (treadmill walking at 60-80% of maximum WS) + upper and lower limb resistance training (set of 10-15 repetitions with weights) + breathing exercises (lip and diaphragmatic breathing) + trunk-centred stretching + patient education (oxygen use, self-management and disease knowledge).<br>A home-based program including aerobic exercise + resistance training + limb/trunk stretching + walking was performed at least 2-3 days/weekly.
**Jackson, 2014 [27]**	Outpatient	12 weeks	2/weekly	Outpatient 2-h session of cardiopulmonary endurance training (20-min of treadmill walking and 10-min of semifrequent cycling at 80% maximum HR) + 15-min flexibility exercises (upper and lower body) + 15-30-min home-based strength training with bands (3 sets of 15 repetitions of upper and lower body) + 1 session biweekly of educational lectures (including medications use, breathing techniques, exercise strategies, proper nutrition, pulmonary physiology, and psychological mechanisms).
**Jarosch, 2020 [28]**	Inpatient	3 weeks	5-6/weekly	A program of medical care (OLT and NIV management) + psychological support + breathing therapy + education (disease management, physical activity, nutritional counseling, and motivation) + 15-18 exercise training sessions (endurance or interval cycling at 60-100% of the peak WR and 3 sets of 15-20 repetitions of resistance training for major muscle groups).
**Kataoka, 2022 [29]**	Combined: supervised and home-based program	52 weeks	12-weeks of 2/weekly inductive outpatient sessions and at least 2/weekly home-based training<br><br>40-weeks of 4/weekly home-based maintenance training and 1/monthly outpatient sessions	A 12-week induction phase of 24 outpatient supervised session of 30-min of endurance (first cycling at 80% of initial WS and after at 80% of the peak WR) + walking and resistance training (upper and lower limbs).<br>A home exercise program was practiced at least 2/weekly.<br>A 40-week maintenance phase of home-based self-training and 1/monthly supervised outpatient training.

**Shen, 2021 [30]**	Home based	12 months	3 times/daily	LHP's RRPF includes 3 consecutive sets of upper body movement, each of one repeated 3 times daily 4-6 times within 1 minute (deep breath of the whole, unilateral lower and upper lung).
**Vainshelboim, 2014 [31]**	Supervised outpatient	12 weeks	2/weekly	Two 6-week block of 60-min sessions. First block: 5-/8-min of calisthenics exercises, stretching and deep breathing exercises + 30-min of aerobic interval training [treadmill walking at 70–80% of WS, cycling at 50–60% of peak WR and up to 15-min of step climbing (5 reps of 5 min bout + 1 min rest) + a 5–8-min bout of self-paced walking + 10- min of upper and lower body resistance training with dumbbells (1 set of 12–15 reps + 1 min rest for 4–6 exercises) + 5-min of flexibility training (1 set of 4–5 stretching for 15–30 s). Second block: 20-min of step climbing + continuous aerobic endurance training (cycling at 60–70% of peak WR and treadmill walking at 80–90% of WS) + 3–5 min stair climbing + resistance (2 sets of 10–12 reps + 45 s rest) and flexibility training (2 sets of 15–30 s).
**Vainshelboim, 2015 [32]**	Outpatient	12 weeks	2/weekly	>>
**Vainshelboim, 2016 [33]**	Outpatient	12 weeks	2/weekly	>>
**Vainshelboim, 2017 [34]**	Outpatient	12 weeks	2/weekly	>>
**Yuen, 2019 [35]**	Home-based	12 weeks	3/weekly	30-min Wii Fit exergame sessions 3/weekly. An additional 30-min physical activity 3/weekly.
**Zhou, 2021 [36]**	Home-based (PD)<br><br>Outpatient (PR)	8 weeks	5/weekly	8-weeks PD program of 2 stages, each made up of 5 sessions/weekly 2 stages: 1-month supervised intensive exercise phase of 1-h exercise time 2/daily + 1-month unsupervised home-based exercise phase.<br><br>2-month PR program of 5-min muscular relaxation, 20-min exercise (cycling at maximum HR) + 10-min relaxation training.

Abbreviations: WR, work rate peak; WS, working speed; MIP, maximum inspiratory speed; HRR, heart rate reserve; HR, heart rate; VAPA; virtual autonomous physiotherapist agent; OLT, long-term oxygen therapy; NIV, non-invasive ventilation; WL, workload; LHP RRF, LHP's Respiratory Rehabilitation for Pulmonary Fibrosis; PD, pulmonary Dayoin; PR, pulmonary rehabilitation
